# The genetic diversity of soil bacteria affected by phytoremediation in a typical barren rare earth mined site of South China

**DOI:** 10.1186/s40064-016-2814-0

**Published:** 2016-07-19

**Authors:** Shenghong Liu, Wen Liu, Miaoxian Yang, Lingyan Zhou, Hong Liang

**Affiliations:** College of Life Sciences, Zhongkai University of Agriculture and Engineering, NO. 501, Zhongkai Road, Haizhu District, Guangzhou, 510225 China; State Key Laboratory for Conservation and Utilization of Subtropical Agro-bioresources, South China Agricultural University, NO. 483, Wushan Road, Tianhe District, Guangzhou, 510642 China

**Keywords:** Rare earth mined zone, Phytoremediation, Soil bacteria, Genetic diversity

## Abstract

The soil bacterial diversity is one of the most important indicators to evaluate the effect of phytoremediation. In this study, the technologies of Sequence-Related Amplified Polymorphism (SRAP) and 16S rRNA gene sequence analysis were used to evaluate the soil bacterial diversity after phytoremediation in a barren rare earth mined area. The results showed that the plant density was remarkably increased after the phytoremediation. The SRAP analysis suggested that the soil bacterial diversity declined dramatically after mining, while increased significantly in second and third year of the phytoremediation. A total of eight bacterial genera were identified by using 16S rRNA gene sequence analysis, with *Arthrobacter* and *Bacillus* as the dominant species before the mining, and *Brevibacillus* as the dominant species after the mining and during the first year of the phytoremediation. The *Bacillus*, which was a dominant type of bacteria before the mining, disappeared after mining and appeared again in the second and third years of the phytoremediation, other bacterial genera present. Principal component analysis and 16S rRNA gene analysis revealed a new bacterial type after phytoremediation that was not existed in the original mined area. The results of the present study indicated that the soil bacterial richness and genetic diversity significantly increased after the phytoremediation in the mined site.

## Background

China is the major rare-earth resource country, where the rare-earth reserves, production, and sales were highest in the world (Wu et al. 2010; Gao and Zhou 2011). However, due to the long-term exploitation, many rare-earth mining areas showed different kinds of problems, such as destroyed soil structure, reduced soil fertility and organic matter deficiency, resulting in soil impoverishment which is very unfavorable for plants growth (Tu et al. 2002; Zhang et al. 2007; Chen 2011; Sun 2014; Zhou et al. 2015). To keep the sustainable development of rare-earth industry, the Chinese State Council Information Office published a white paper *The Status and Policy on the Rare*-*earth Resource of China* in June, 2012, which declares to strengthen the protection against rare-earth resources and environment. In April 2014, the World Trade Organization (WTO) has rebuffed China for the restriction on the export of minerals, which indicated a great challenge for China to conserve the rare-earth resources. The ecological recovery of barren area of the mined sites had become an important task for the sustainable development of rare-earth mining industry in China.

As the rare-earth resources are relatively limited, the remediation schemes for the barren area of the mined sites are also limited. In recent years, a series of studies had been carried out on the impact of phytoremediation on the rare-earth mined areas of the south China. These studies were focused on the factors affecting the plant growth, such as heavy metals contents, physicochemical properties and nutrient contents of soil in the barren areas of mined sites. The phytoremediation studies were conducted on the barren area of the rare earth mined sites in Heping County of Guangdong Province (Liu et al. 2013), Changting County of Fujian Province (Jian 2012), Xinfeng County of Jiangxi Province (Li 2014), and Mianning County of Sichuan Province. These studies have screened different plants species suitable for the mine tailings sites, and developed some remediation schemes. Some of the studies investigated the effects of various soil stress factors on the growth of *Paspalum conjugatum* (Yang et al. 2014), the tolerance of *Sorghum bicolor* to rare-earth elements (Guo et al. 2013), as well as the effects of *Arbuscular mycorrhizae* on reducing the toxicity of rare-earth elements and heavy metals and to facilitate plant growth (Chen and Zhao 2007). In these studies, phytoremediation strategies were established through field experiments or simulation experiments, and the soil structure and physiochemical properties were analyzed. However, there is little information about the effects of phytoremediation under multi-year field conditions. In addition, the change of soil bacterial community was rarely studied in the barren area of the mined sites, which greatly limited the use of phytoremediation technologies for the improvement of rare-earth mined areas.

Bacteria community would be changed due to the change of physicochemical properties and destroy of soil ecosystem after mining actives (Patel and Behera 2011; Shu et al. 2015). Bacteria genetic diversity is related to the vegetation coverage on soil (Xu et al. 2007; Shi et al. 2010), and genetic diversity of bacteria is used as an indicator to measure the effect of phytoremediation. Thus, Analysis of bacteria diversity is important when the soil ecosystems respond to change by phytoremediation (Bond et al. 2000). Randomly amplified polymorphic DNA (RAPD), Amplified fragment length polymorphism (AFLP), Sequence-Related Amplified Polymorphism (SRAP) and 16S rRNA were the commonly used techniques for the mining site bacteria genetic diversity analysis. Among them, the SRAP and 16S rRNA technologies are more precise and inexpensive (Li et al. 2011; Pandey et al. 2013). 16S rRNA gene sequencing technique is applicable in detecting the bacterial phylogenetic relationships and identifying the unknown bacteria through sequence alignment in Genbank (Xiong et al. 2011; Zhou et al. 2015). With the development of nucleotide sequencing technology, 16S rRNA gene sequences of more and more microorganisms were sequenced and included into the international public databases, which makes 16S rRNA gene sequences analysis a more convenient tool for the species identification as well as community analysis of bacteria (Chen et al. 2006; Lu et al. 2015). SRAP molecular markers analysis technology, which characterized by simple operation, abundant polymorphism, good repetition as well as high sensitivity, is a general technique for the soil bacterial biodiversity analysis (Zhang et al. 2012). Thus, these two technologies can provide an good insight into the successional ecological changes during the process of phytoremediation.

Present study evaluated the changes of bacterial diversity in the rare earth mined sites before and during the phytoremediation (consecutive 4 years from 2009 to 2012), by using SRAP markers and 16S rRNA sequencing method, aiming to investigate the effects of phytoremediation on the soil bacteria diversity in the barren rare earth mined site.

## Methods

### Phytoremediation and soil collection

The study was processed in 815 mining site, Xiache Town, Heping County, Guangdong province, it is a typical ion-absorbed rare-earth mining area in South China. The mining was started in the 1980s and ended in 2005. The excessive mining had led to severely impacted mine tailings and was characterized by water and soil loss, as well as high levels of heavy metals contaminants, landslide, and ecological environment destruction of the mountains.

The phytoremediation was carried out during 2009 to 2012. The barren area of the mined site was first applied with dried poultry manure at the rate of 150 kg per 100 m^2^ in June 2009. Then, the seeds of *Stylosanthes scabra*, *Tephrosia candida*, *Cajanus cajan*, *Medicago sativa*, *Vetiveria zizanioides*, *Digitaria sanguinalis* and *Paspalum conjugatum* were sown by broadcasting in the site, and let them grow naturally. The photographs of repaired region were taken in June of each year during 2009 to 2012.

A total of five samples were collected from the rare-earth mined site in the mid of June of each year during 2009 to 2012, with one sample per year, and the soil from the unexplored area nearby as a control. The total sampling area is 1000 m^2^, with one sampling point was set per 30 m^2^. For each point, 500 g of the soil in a depth of 0–20 cm was collected with a sterilized spatula. The soil samples from different locations in the same year were mixed thoroughly, packed in sterile plastic bags and then, transported in a foam box fill with ice to the laboratory of Zhongkai University of Agriculture and Engineering in Guangzhou city for isolation and culturing of bacteria.

### Bacterial isolation and DNA extraction

The soil samples (0.5 g) were put into 10 mL centrifuge tubes respectively and suspended in 5 mL of double-distilled water (ddH_2_O). The suspension of 200 µL of each sample was coated evenly in the Luria–Bertani Broth (LB) solid medium (the common medium for soil bacteria culturing, also named Lysogeny broth), and cultured in the petri dishes at 37 °C for 12–24 h (Zhang et al. 2007). Twenty-four single colonies were selected from each sample, and a total of 120 single colonies were selected from five soil sample, and cultured in the LB medium at 37 °C for 12–24 h. Then, the bacterial DNA was extracted according to the modified protocol of Liu et al. (2008). Briefly, the DNA of soil bacteria was extracted by SDS extracting solution with high salty and low pH modifications, and purified with phenol: chloroform: isopentanol (25:24:1), followed by washing with cold 70 % ethanol, air dried and subsequently resolved in TE buffer (10 mmol/L Tris and 0.1 mmol/L EDTA). Then the DNA was equilibrated to 25 ng/mL for SRAP analysis.

### SRAP analysis

The primers used for SRAP analysis were selected from the previously published data (Li et al. 2011). We selected six primers pairs, including F1R3, F1R8, F2R3, F2R4, F5R3 and F5R7 in the present study (Table 1). The 25 µL PCR reaction mixture was consisted of 10 ng template, 0.15 µmol/L primer pairs, 1.0 µL dNTPs (2.0 mmol/L each), one unit Taq polymerase, and 1 × PCR buffer (50 mmol/L KCl, 10 mmol/L Tris–HCl pH 8.3, 1.5 mmol/L MgCl_2_, 0.01 % glatin). The PCR reaction profile was as follows: 94 °C for 5 min, 1 cycle; 94 °C for 45 s, 35 °C for 1 min, 72 °C for 90 s, 5 cycles; 94 °C for 45 s, 50 °C for 1 min, 72 °C for 90 s, 35 cycles; an additional extension at 72 °C for 10 min, followed by holding at 4 °C after the reaction.

**Table 1:** Primers used in the present study

Process	Primers name	Sequence	Primers name	Sequence
SRAP	F1	TGAGTCCAAACCGGATA	R3	GACTGCGTACGAATTGAC
	F2	TGAGTCCAAACCGGAGC	R4	GACTGCGTACGAATTTGA
	F5	TGAGTCCAAACCGGAAG	R7	GACTGCGTACGAATTCAA
			R8	GACTGCGTACGAATTAGC
16S rRNA gene	16S-F	AGAGTTTGATCATGGCTCAG	16S-R	GGTACCTTGTTACGACTT

The products of PCR reaction was detected by electrophoresis, using 0.1 % AgNO_3_ as stain solution, and 400 mL of aqueous solutions containing 0.076 g sodium borate, 6 g NaOH and 1.6 mL formaldehyde as the image developer (Liu et al. 2015). The developed films were photographed in a Gel Imaging System and Genetools software (SynGene) under white light.

### Bacterial 16S rRNA gene amplification and sequencing

Primers used for 16S rRNA gene amplification are shown in Table 1 (Pandey et al. 2013). The 50 µL PCR reaction mixture was consisted of 20 ng template, 0.2 µmol/L primer pairs, 2.0 µL dNTPs (2.0 mmol/L each), two unit Taq polymerase, and 1 × PCR buffer (50 mmol/L KCl, 10 mmol/L Tris–HCl pH 8.3, 1.5 mmol/L MgCl_2_, 0.01 % glatin). The PCR cycling procedure was as follows: Initial denaturation at 94 °C for 4 min, followed by 30 cycles of denaturation at 94 °C for 45 s, annealing at 57 °C for 45 s, and extension at 72 °C for 2 min, and an additional extension at 72 °C for 10 min. Then, the PCR samples were hold at 4 °C after the completion of reaction. The PCR product of 3 μL was detected with electrophoresis in 1.5 % agarose gel. The PCR products with clear single target band were sequenced by Invitrogen Co. Ltd.

### Data analysis

The markers polymorphisms was scored according to the method of Kuroda et al. (2009). The gel bands were transformed into an original data matrix, in which the locus with band was assigned as “1” and that without band was assigned as “0”. Percentage of polymorphic bands (PPB) = The number of polymorphic bands/Total number of bands × 100 %. The average number of alleles (na), number of effective alleles (ne), Nei’s gene diversity index (h), Shannon’s diversity index (I), Nei’s genetic identity and the genetic distance were calculated by using POPGENE 32 software; The Polymorphism information content (PIC) was calculated by Piccal software (Version_0.6, Yellow Sea Fisheries Research Institute, Chinese Academy of Fishery Sciences, 2007) using the formula: ${\text{PIC}} = 1 - \sum\nolimits_{i = 1}^{l} {P^{2}_{i} - \sum\nolimits_{i = 1}^{l - 1} {\sum\nolimits_{l = i + 1}^{l} {2P^{2}_{i} P^{2}_{j} ,P_{i} \;{\text{and}}\;P_{j} } } }$ (are the population frequency of the *i*th and *j*th allele) (Bosttein et al. 1980; Liu et al. 2015). The cluster analysis was performed by using NTSYS2.10 software according to the intra-population Nei’s genetic identity. The principle component analysis was done by using SPSS 17.0 software (Jin et al. 2013). The 16S rRNA gene sequences were assembled using DNA Star software. The bacterial species and genus were identified by homology analysis using Blast tool in NCBI database.

## Results

### Phytoremediation of the mined site

**Fig. 1:**
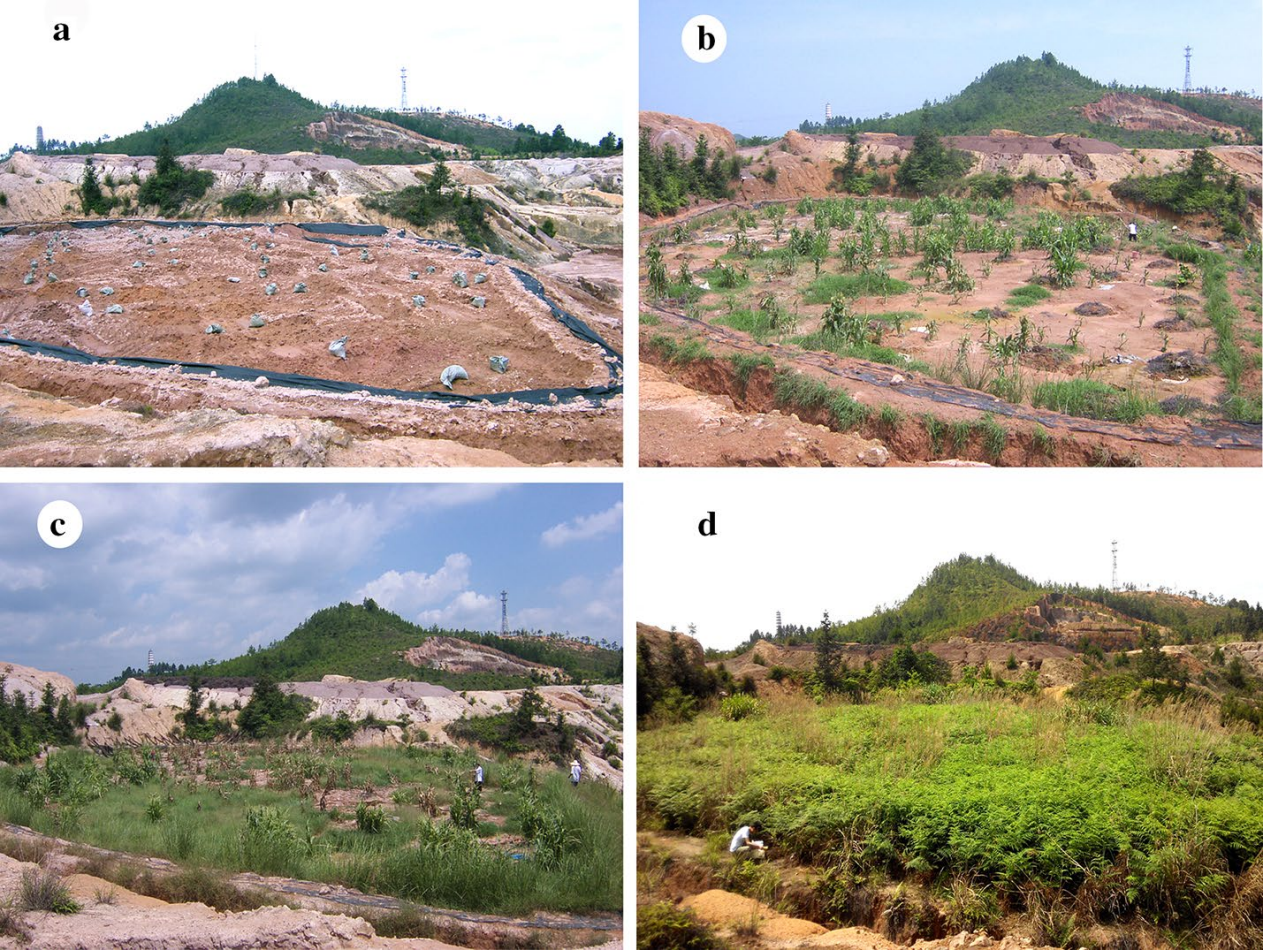
The effects of the phytoremediation in the barren rare-earth mined site. The *photo* shows the 815 rare earth mined area in 2009 (**a**), 2010 (**b**), 2011 (**c**) and 2012 (**d**), respectively

As the rare-earth was extracted using ammonium sulfate extraction method in the 815 rare-earth mining area, the soil structure and composition were severely damaged and there was almost no plant in the site. The long term soil bareness led to serious soil and water loss (Fig. 1a). In present study, through the application of dried poultry manure following by the seeding with seven plants, the vegetation cover remarkably increased in the rare-earth mined area year by year. The vegetation covered the whole experimental mined site during the third year of phytoremediation, and the ecological conditions improved significantly (Fig. 1b–d).

### Genetic diversity analysis of soil bacteria by SRAP

**Fig. 2:**
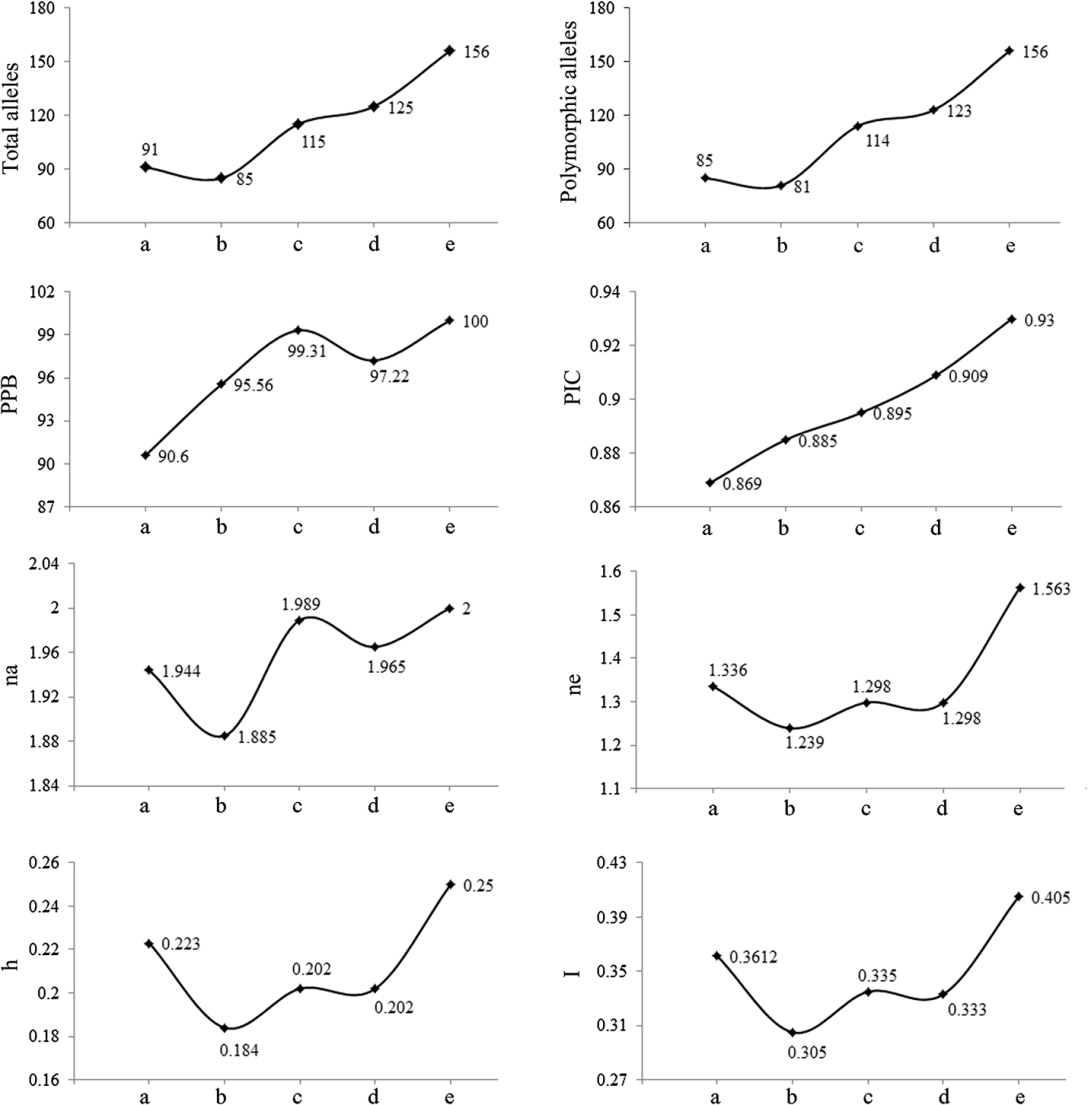
The annual variation in the soil bacteria detected by SRAP markers in the rare-earth mined site. *a* Before mining, *b* after mining, *c* first year of phytoremediation, *d* second year of phytoremediation, *e* third year of phytoremediation

A total of 120 single colonies from five soil sample were used to bacteria genetic diversity analysis. We detected significant changes in the eight parameters of soil bacterial genetic diversity by using six pairs of SRAP primers. The total loci, polymorphicloci, na, ne, h and I values significantly decreased after the mining. All the genetic diversity parameters detected gradually increased year by year after the phytoremediation. Among them, the values for total loci, polymorphic loci and na were exceeded that before mining in the first year of phytoremediation, and the Ne, h and I values were exceeded that before mining in the third year of phytoremediation. The percentage of polymorphism bands (PPB) and the polymorphism information content (PIC) values were also increased year by year. These results indicated that the soil bacterial community in the rare-earth mined area decreased significantly after mining, and increased remarkably after the phytoremediation, even exceeded that before the ore processing (Fig. 2).

**Fig. 3:**
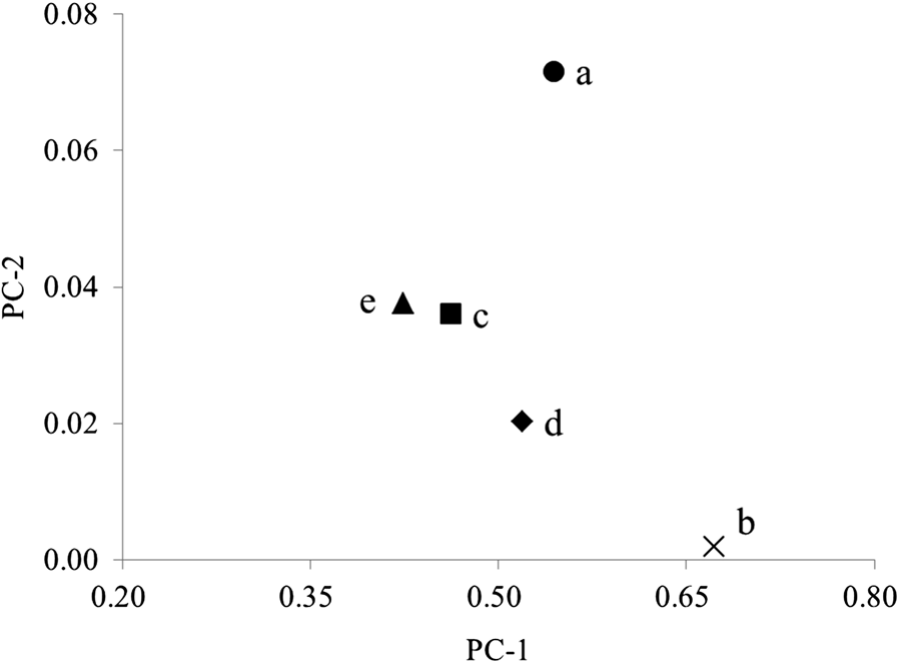
Relationship of five soil bacterial community by principal components analysis. *a* Before mining, *b* after mining, *c* first year of phytoremediation, *d* second year of phytoremediation, *e* third year of phytoremediation; PC-1, the first principle component; PC-2, the second principle component

To understand the changes of soil bacterial communities in different stage of phytoremediation, PCA analysis was carried out (Fig. 3). The results showed that the principle components of bacterial communities changed significantly after mining, indicating the significant impact of mining on the soil bacteria. The principal components of bacterial communities of samples from different stage of phytoremediation were similar between each other, and were close to that from unexploited site, while significantly different from the mined site before phytoremediation. These indicated that the soil would be further restored by phytoremediation.

**Fig. 4:**
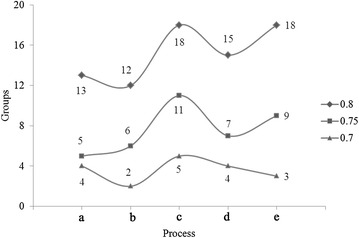
The group variation in the rare-earth soil bacterial community under different genetic coefficient thresholds. *a* Before mining, *b* after mining, *c* first year of phytoremediation, *d* second year of phytoremediation, *e* third year of phytoremediation

According to the genetic coefficient of phylogenetic tree, the soil bacteria can be divided into different groups by genetic coefficient thresholds. Under the genetic coefficient thresholds of 0.70, 0.75 and 0.80, the soil bacteria species decreased after mining, and increased to certain extent after phytoremediation (Fig. 4). We calculated the Nei’s genetic identity and genetic distance of the five samples, and found that the genetic distance was significant changed among five bacterial communities (Table 2).

**Table 2:** Nei’s genetic identity and genetic distance among five bacterial communities

Process	a	b	c	d	e
a	****	0.880	0.902	0.859	0.884
b	0.128	****	0.879	0.869	0.884
c	0.103	0.129	****	0.890	0.900
d	0.152	0.140	0.116	****	0.870
e	0.123	0.124	0.105	0.139	****

The data in the upper half of the table were the Nei’s genetic identity, and those in the bottom half of the table were the genetic distance

a, before mining; b, after mining; c, first year of phytoremediation; d, second year of phytoremediation; e, third year of phytoremediation

**Fig. 5:**
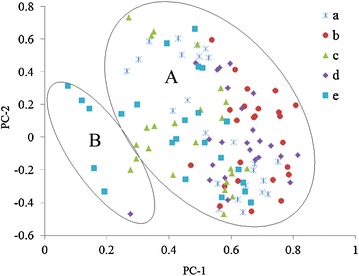
The distribution of soil bacterial community detected by using principal components analysis. *a* Before mining, *b* after mining, *c* first year of phytoremediation, *d* second year of phytoremediation, *e* third year of phytoremediation; PC-1, the first principle component; PC-2, the second principle component

The soil bacteria from soil samples can be divided into two groups on the basis of principle component analysis. The first group consists of the soil bacteria found in all samples from one to three year phytoremediation, which accounted for most of the soil bacteria (Fig. 5, A group). The second group contained two soil bacteria detected in the soil from the first year of phytoremediation, one soil bacteria found in the soil from the second year phytoremediation and five soil bacteria detected in the soil from the third year of phytoremediation (Fig. 5, B group). These results indicated that new bacterial species occurred after the phytoremediation, which might be introduced into the site with the plants or the manure.

### 16S rRNA gene polymorphism of the soil bacteria

A total of eight bacteria genera were identified in the soil samples from the rare-earth mining area by using 16S rRNA polymorphism analysis. There was two bacterial genera (*Arthrobacter* and *Bacillus*) detected in the unexploited area, while only one genus (*Brevibacillus*) was found after the mining and during the first year of phytoremediation. The number of bacterial genera increased to four (*Bacillus, Bacterium*, *Lysinibacillus*, and *Sinorhizobium*) and five (*Paenibacillus*, *Bacillus*, *Sinorhizobium*, *Solibacillus* and *Bacterium*) in the second and third year of phytoremediation, respectively (Table 3). These results suggested that the rare-earth mining changed the soil environment and natural habitat of the bacteria, and led to the disappearance of some original soil bacteria. Phytoremediation significantly increased the number of soil bacteria species during the 3 years of field study. Thus, these results showed that phytoremediation was beneficial for maintaining the diversity of the soil bacterial community.

**Table 3:** The identification of soil bacteria based on16S rRNA gene

Process	*Arthrobacter*	*Bacillus*	*Brevibacillus*	*Bacterium*	*Lysinibacillus*	*Sinorhizobium*	*Paenibacillus*	*Solibacillus*
a	15	9	–	–	–	–	–	–
b	–	–	24	–	–	–	–	–
c	–	–	24	–	–	–	–	–
d	–	19	–	2	2	2	–	–
e	–	11	–	–	–	4	8	2
Most similar accessions	AB588633.1	AB112727.1	AB112730.1	AH003322.1	AM903104.1	AM935933.1	FR728383.1	NR074954.1

–, not detected

a, before mining; b, after mining; c, first year of phytoremediation; d, second year of phytoremediation; e, third year of phytoremediation

Before the mining, the dominant genus was *Arthrobacter* and *Bacillus*, while *Brevibacillus* become the dominant genus after the mining and during the first year of phytoremediation. In the second and third year of phytoremediation, *Bacillus*, the dominant genus before the mining, was detected again in the soil, which had 19 and 11 species in the second and third year of phytoremediation, respectively (Table 3). These results indicated that the *Bacillus* was the dominated type in the favorable eco-environmental conditions, while *Brevibacillus* was the most frequent type in the adverse eco-environmental conditions.

## Discussion

The ecological factors in the rare-earth mined areas, such as soil physicochemical properties and climatic conditions changed significantly after the mining (Li 2006). The mined areas are usually lack of nutrients (N, P, K) and organics, and with poor physical structure and extreme pH (Rotkittikhun et al. 2006; Mendez et al. 2007; Mendez and Maier 2008). Thus, the most of the plants disappeared due to the adverse growing conditions. Like these previous studies, the soil at the rare earth mining land in present study was bare and deserted after mining, organic and nutrients components were decreased with the bacteria reduction by rain leach (Li et al. 2013). Naturally, it will take a long time for the vegetation establishment in the barren mined site because of the poor plant diversity, low plant biomass and the unfavorable plant growing conditions (Zhou et al. 2015). By artificial phytoremediation for 3 years, the plant coverage greatly increased, and the soil fertility was in present study was greatly improved with organic and nutrients content more than ten times higher than that before (Li et al. 2013).

In consistent with the soil physiochemical property improvement and vegetation coverage increase, we found that the genetic diversity of bacteria was poor in the mined barren land, while significant improved after phytoremediation and even exceeded that before mining. In one hand, the plants and insects might bring new bacteria into the land. In another, the metabolism of plants improved permeability of the soil, and provided an environment rich in organics for microbial. The soil rich in organics has higher microbial diversity (Bossio and Scow 1998; Li and Quiros 2001; Zhong and Cai 2004). In contrary, the bacteria activity helped to improve the soil environment for plants to grow. The soil microorganisms are involved not only in the formation and degradation of soil humus, but also in the transformation and recycling of soil nutrition. Soil microorganisms play key role in the degradation of animals and plants residues, and thus promote the mass cycling and energy fluxes. The distribution and activity of the microorganisms in the soil intimately affects the soil structure formation and the nutrition transformation (Mendez et al. 2007). The soil microorganisms are able to improve the soil fertility by mineralizing the organic matters, and transform the insoluble organic matters into soluble matters that can be utilized directly by plants. Thus, the subtle interaction between the plants, bacteria and soil components might contribute to the improvement of the ecosystem in the mined barren land in present study.

Soil bacteria are getting more and more attention in the soil quality evaluation as one of the most important indicator of the soil quality and soil ecological function (Olsen et al. 1986; Liu et al. 2012; Olivera et al. 2016). In present study, the bacteria species changed after mining. By phytoremediation, some of the bacteria re-emerged, while some were not, and new species occurred. Before mining, *Arthrobacter* and *Bacillus* were the dominant species, while the *Brevibacillus* become the dominant species after mining. *Bacillus* disappeared after mining and re-emerged in the second and third years of the phytoremediation, while *Arthrobacter* was not found after phytoremediation. Principal component analysis and 16S rRNA gene analysis revealed a new bacterial type after phytoremediation that was not existed in the original mined area. These were in consistent with the previous studies, that the number of bacteria would decrease obviously after mining, although the nutrients and organics were restored, the physical structure and most of the plants were different from that before mining (Mendez et al. 2007; Zhou et al. 2015). As chemoheterotrophic bacteria (Samanta et al. 1999), *Arthrobacter* would be sensitive to the changes of soil contents, structure and biology components, this might be the reason that it was isolated only from soil before mining in present study. The soil showed specific physicochemical and biological characteristics in different stage of phytoremediation, which might explained why some bacteria (such as *Lysinibacillus*, *Paenibacillus* and *Solibacillus*) were isolated only from second or 3 years of phytoremediation.

The results of present study demonstrated the reliable application of 16S rRNA gene sequence analysis and the SRAP molecular markers analysis technology to detect the biodiversity in soil microbial community in rare-earth mined area. Here, the genetic diversity and genera of microbial community were shown to be affected by mining and phytoremediation. The biodiversity of soil bacteria significantly decreased after the mining, and increased annually with the application of phytoremediation. According to previous studies, there were different media which would be optimum for the isolation of different groups of bacteria (Yadav et al. 2015a, b; Verma et al. 2016). In present study, we used LB medium, which was suitable for the culturing of most soil bacteria, to isolate the bacteria from five soil samples under the same nutrient and physics properties.

It is worth to mention that, in the second and third year of phytoremediation, the coverage of vegetation in rare earth mining site was improved, and the soil bacteria biodiversity had even exceed that before mining, which indicated that phytoremediation was not only beneficial in recovering the soil environment, but also beneficial in maintaining a high bacterial biodiversity.

## Conclusions

This study demonstrated the plant coverage was remarkably increased after the phytoremediation in the barren land of a rare earth mined site. The soil bacterial genetic diversity decreased after the mining, while significantly increased annually with the ecological restoration. It is worth to mention that, in the second and third year of phytoremediation, the vegetation coverage in rare earth mining site was improved obviously, and the soil bacterial biodiversity had even exceeded that before mining, these indicated that the bacteria genetic diversity was enriched by phytoremediation.
